# Single-step fabrication and work function engineering of Langmuir-Blodgett assembled few-layer graphene films with Li and Au salts

**DOI:** 10.1038/s41598-020-65379-1

**Published:** 2020-05-21

**Authors:** Ivana R. Milošević, Borislav Vasić, Aleksandar Matković, Jasna Vujin, Sonja Aškrabić, Markus Kratzer, Thomas Griesser, Christian Teichert, Radoš Gajić

**Affiliations:** 1 Laboratory for Graphene, other 2D Materials and Ordered Nanostructures of Center for Solid State Physics and New Materials, Institute of Physics, University of Belgrade, Pregrevica 118, 11080 Belgrade, Serbia; 2 Institute of Physics, Montanuniversität Leoben, Franz Josef Str. 18, 8700 Leoben, Austria; 3 Nanostructured Matter Laboratory of Center for Solid State Physics and New Materials, Institute of Physics, University of Belgrade, Pregrevica 118, 11080 Belgrade, Serbia; 4 Institute of Chemistry of Polymeric Materials, Montanuniversität Leoben, Otto-Gloeckel-Straße 2, 8700 Leoben, Austria

**Keywords:** Materials science, Nanoscience and technology

## Abstract

To implement large-area solution-processed graphene films in low-cost transparent conductor applications, it is necessary to have the control over the work function (WF) of the film. In this study we demonstrate a straightforward single-step chemical approach for modulating the work function of graphene films. In our approach, chemical doping of the film is introduced at the moment of its formation. The films are self-assembled from liquid-phase exfoliated few-layer graphene sheet dispersions by Langmuir-Blodgett technique at the water-air interfaces. To achieve a single-step chemical doping, metal standard solutions are introduced instead of water. Li standard solutions (LiCl, LiNO_3_, Li_2_CO_3_) were used as n-dopant, and gold standard solution, H(AuCl_4_), as p-dopant. Li based salts decrease the work function, while Au based salts increase the work function of the entire film. The maximal doping in both directions yields a significant range of around 0.7 eV for the work function modulation. In all cases when Li-based salts are introduced, electrical properties of the film deteriorate. Further, lithium nitrate (LiNO_3_) was selected as the best choice for n-type doping since it provides the largest work function modulation (by 400 meV), and the least influence on the electrical properties of the film.

## Introduction

Graphene, consisting of a single layer carbon arranged in a hexagonal lattice, has attracted extensive interest because of the excellent mechanical and electrical properties associated with its two dimensional structure^1–4^. Chemical vapor deposition (CVD) method has become the most common method for production of large-area graphene films^5^. Still, simple and low-cost methods are needed for mass production especially when considering the cases where high-quality films are not needed for the desired functionality, as in low-power lighting, sensors, transparent heating, and de-icing applications^6^. In that context, liquid-phase exfoliation (LPE) is a perspective way of obtaining large quantities of exfoliated graphite in solution. LPE of graphite results in a dispersion of few-layer graphene sheets (GSs) in the solvent. However, in order to access the full potential of LPE-processed graphene, thin-films needs to be controllably fabricated utilizing techniques capable to introduce self-ordering of GSs^7^. One such example is Langmuir-Blodgett assembly (LBA). Based on surface-tension induced self-assembly of nanoplatelets at the liquid-air interface or the interface of two liquids, LBA is a good method for production of large-scale, highly transparent, thin solution-processed graphene films^8–11^.

Excellent electrical conductivity, flexibility and transparency in the visible domain make graphene a natural choice for ultrathin, flexible and transparent electrodes in electronic devices^10,12–19^. Still, a significant work function difference between graphene and frequently employed active layers of photovoltaic and light-emitting diode (LED) devices gives rise to a high contact resistance. Contact resistance can have a significant impact on overall efficiency and performance of the devices^20^. This is of a particular technological relevance considering that any realistic application of graphene based transparent electrode must compete against those based on indium tin oxide (ITO) or fluorine-doped tin oxide (FTO), which have already gone through decades of interfacial optimization in order to deliver todays’ performance^21–23^. Therefore, the understanding of the efficient ways for modulation of the graphene work function is crucial for improving device performances^21,22,24^. In order to enhance the charge injection, the work function of the graphene electrode should be optimized to better match WF of the adjacent layer in order to form an ohmic contact^24^.

Recently, the chemical doping has been reported to be an effective method for doping of CVD graphene and tuning its work function by charge transfer between the graphene sheet and metal salts, organic dopants, or metal oxide layers^12,14,21–28^. Such surface charge transfer induced by chemical doping is expected to efficiently control the Fermi level of graphene sheets without introducing substitutional impurities or basal plane reactions, thus, preventing any damage to the carbon networks and not introducing scattering centres that would lower carrier mobility^21^. Kwon *et al*. reported n-type chemical doping of CVD graphene with alkali metal carbonates by soaking in appropriate solutions^23^ and alkali metal chlorides by spin-coating of appropriate solutions on the transferred graphene substrates^25^. So far, doping of Langmuir-Blodgett graphene films prepared from LPE dispersions has been done with nitric acid and ozone after the film was formed using the drop-casting method and UV/ozone treatment^9,29^. Chemical doping is especially attractive for LPE-based graphene films since many exposed edges of GSs are expected to enable very efficient functionalization through charge transfer doping. However, the chemical doping with metal salt solutions has not been used to control the work function of LBA graphene films so far. In this work LBA graphene films obtained from LPE dispersion were doped during the process of film formation. Therefore, the formation and doping of the LBA graphene films in our work represent a single-step process. This is a significant improvement compared to previous works where the chemical doping was applied only after the graphene fabrication.

In the present work, we systematically investigated single-step work function modulation (increase and decrease) of the LPE GS films achieved by chemical doping. In particular, using Li standard solutions (LiCl, LiNO_3_ and Li_2_CO_3_) as n-dopant, and gold standard solution H(AuCl_4_) as p-dopant was investigated. In contrast to previous methods for chemical doping of CVD graphene which can be applied only after the graphene films fabrication, here we described the method for the production and doping of LPE graphene films in a single-step. Single-step work function modulation means doping of the film at the moment of its formation from the LPE graphene dispersion by LBA technique at the air-metal standard solution interface. We have demonstrated tunability of the WF in the range of almost 1 eV, making these metal-salt treated LPE-based graphene electrodes suitable candidates for both electron and hole injection interfaces.

## Results and discussion

### Morphology of LPE GS films

**Figure 1:**
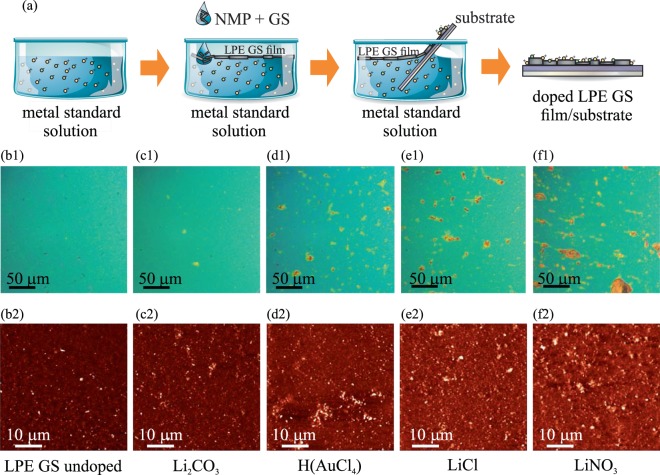
(**a**) Schematic representation of the LPE GS film formation and its doping in the single-step process. (**b1**–**f1**) Optical images are shown in the top row, whereas (**b2**–**f2**) AFM topographic images are shown in the bottom row for the following cases: (**b**) undoped LPE GS film, and (**c**) Li_2_CO_3_, (**d**) H(AuCl_4_), (**e**) LiCl, (**f**) LiNO_3_ doped LPE GS films. z-scale in all AFM images is 100 nm.

Fabrication and doping of the GS films is schematically represented in Fig. 1(a): air-metal standard solution interface, introduction of LPE dispersion and formation of the LPE GS film at the interface, scooping of the doped film on the target substrate and finally, obtained doped LPE GS film which is further investigated with different techniques.

Morphology of LPE GS films is depicted in Fig. 1 consisting of both optical (Fig. 1(b1-f1)) and Atomic Force Microscopy (AFM) topographic images (Fig. 1(b2-f2)) for both undoped and metal doped LPE GS films. As can be seen from AFM images, the doping process does not change morphology of LPE films, except that the doped films contain more agglomerates (visible as bright particle-like domains). The following values for the surface roughness were obtained by AFM measurements averaged on ten 50  ×  50 µm^2^ areas: (a) 11.9 ± 1.5 nm for undoped LPE GS film, (b) 11.5 ± 3.5 nm for Li_2_CO_3_ doped, (c) 13.3 ± 2 nm for H(AuCl_4_) doped, (d) 13.7 ± 1.6 nm for LiCl doped, and (e) 13.8 ± 1.2 nm for LiNO_3_ doped LPE GS films. Therefore, the surface roughness sligtly increases by around 2 nm after the doping, while for Li_2_CO_3_ doped LPE GS film is practically the same as for the undoped film. Still, optical images recorded on larger scale depict formation of agglomerates in doped films which could degraded their optical (leading to an increased scattering and/or absorption of incoming lights on these clusters) and electrical properties (due to enhanced scattering of charge carriers).

The observed formation of the agglomerates is most likely not an inherent property of the particular metal-salt doping. Overcoming this would likely require further optimization of the LBA process. However, as a benchmark the LBA process in this study was optimized for an undoped film and was left unchanged for all of the metal-salt doped films.

### Transmittance measurements

**Figure 2:**
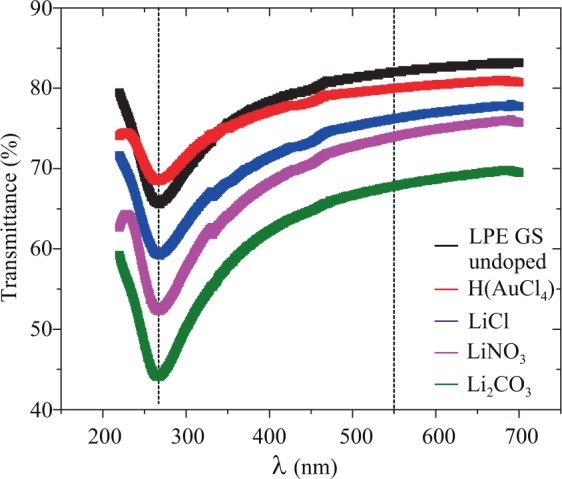
Transmittance spectra of undoped and H(AuCl_4_), LiCl, LiNO_3_, Li_2_CO_3_ doped LPE GS films.

Using the different doping metal standard solution during LBA of graphene films was found to result in different transparency. In the UV region, the transmittance of graphene is dominated by an exciton-shifted van Hove peak in absorption^9,30^. Transmittance at 550 nm was 82% for undoped and 80%, 76%, 74%, 68% for H(AuCl_4_), LiCl, LiNO_3_, Li_2_CO_3_ doped LPE GS films, respectively (Fig. 2). It can be seen that transmittance decreases for doped LPE GS films. Metal salts decrease the transmittance of the graphene films regardless the type of the present metal (gold or lithium). The degree of the transmittance decrease was related to not only the metal cations but also the anions. Different lithium salts decrease transmittance in different amounts. Transmittance decrease of 14% was the highest for the LPE GS film doped with lithium carbonate (Li_2_CO_3_). Similar results of the transmittance decrease for metal doped CVD graphene films were obtained in studies of Kwon *et al*.^22,23,25^. Transmittance decrease could be a consequence of the metal particles adsorption and agglomeration on doped films after the solvent evaporation process. Changes in the thickness of LPE GS films with doping could not be excluded because LBA process in this study was optimized for an undoped film and was left unchanged for all of the metal-salt doped films.

### Raman measurements

**Figure 3:**
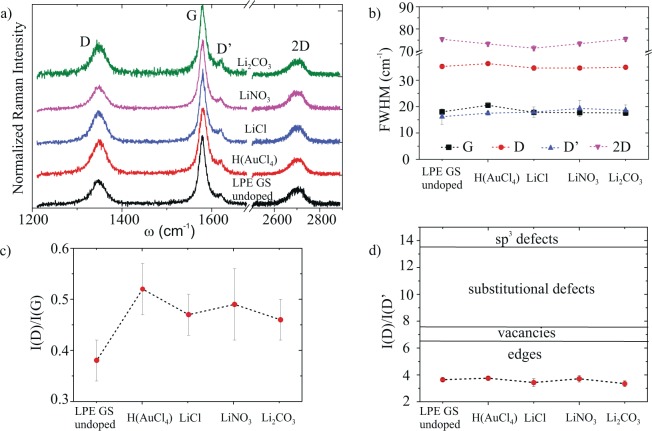
(**a**) Raman spectra of the undoped LPE GS film and films doped with Li and Au salts, (**b**) FWHM of the four basic Raman peaks (**c**)The intensity ratio of D to G peak for different doping metal salts, I(D)/I(G), (**d**) The intensity ratio of D to D’ peak, I(D)/I(D’), for different doping metal salts. We refer to peak intensity as the height of the peaks as proposed by Eckmann *et al*.^32^.

Raman spectra for undoped and H(AuCl_4_), LiCl, LiNO_3_, Li_2_CO_3_ doped LPE GS films are given in Fig. 3(a). The four basic graphene/graphite peaks D (~1348 cm^−1^), G (~1579 cm^−1^), D^’^ (1614 cm^−1^) and 2D (2700 cm^−1^) are observed for all the samples. No significant shifts of any characteristic Raman peaks of graphene were detected after chemical doping (Fig. 3(a)).

The change of the full weight at half-maximum (FWHM) of the Raman modes after doping with metal standard solutions was negligible Fig. 3(b). The only notably change of the Raman spectra was the increase of the intensity ratio of D to G peaks, I(D)/I(G) (Fig. 3(c)). The quantity of defects has been shown to be related to the ratio between the D and G peaks, I(D)/I(G); the larger the ratio, the larger the defect density^31^. We observe increase of the defect density with H(AuCl_4_), LiCl, LiNO_3_, Li_2_CO_3_ doping in relation to the undoped film and the amount of the increase expressed in percent was 37%, 24%, 29% and 21%, respectively.

All self-assembled films suffer from a large defect density that often leads to a high sheet resistance of deposited film. Therefore, the nature and density of defects in any thin film transparent conductor is important, especially when chemical treatment was used to enhance films’ performance. The intensity ratio between the D and D’ peak can be used to get information on the nature of defects in graphene^32,33^. I(D)/I(D’) was calculated, and the obtained results were presented in Fig. 3(d). Topological defects (like pentagon-heptagon pairs), boundaries, vacancies, substitutional impurities and sp^3^ defects are possible defects in graphene^31^. Studies reporting a ratio of 3.5 for boundaries, 7 for vacancies, 13 for sp^3^ and values in-between those for vacancies and sp^3^ for substitutional impurities can be found in the literature^31,32,34^. From Fig. 3(d) it can be observed that the D to D’ intensity peak ratio is nearly constant in our samples regardless of the doping solution, and the value of the ratio indicates that the edges are the dominant type of defects in our LPE GS films.

### Fourier transform infrared absorbance (FT-IR) measurements

**Figure 4:**
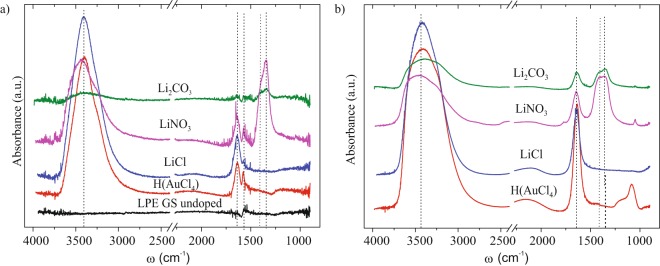
FT-IR spectra of (**a**) undoped and H(AuCl_4_), LiCl, LiNO_3_, Li_2_CO_3_ LPE GS doped films, (**b**) metal standard solutions (0.1 mg/mL) used for doping processes.

FT-IR spectra of undoped and LiCl, LiNO_3_, Li_2_CO_3_, H(AuCl_4_) doped LPE GS films, as well as FT-IR spectra of corresponding metal standard solutions are shown in Fig. 4.

For the undoped LPE GS film FT-IR spectra is simple. It can be seen only a small peak assignable to C=C skeletal vibration^35–37^ of the graphene basal planes at ~1560 cm^−1^. This peak can also be seen in FT-IR spectra for all investigated doped films at the same wavenumber indicating that graphene basal planes were not interrupt by doping. The strong peak at around ~3400 cm^−1^ and another, smaller one, near ~1630 cm^−1^ can be seen in all doped LPE GS films (Fig. (4a)) and corresponding metal standard solutions (Fig. (4b)). They are attributed to the water molecules and are assignable to the O-H stretching vibrations (~3400 cm^−1^) and H-O-H bending mode (~1630 cm^−1^)^38,39^. In the case of FT-IR spectra for LPE GS film doped with LiNO_3_ the peak at ~1340 cm^−1^ and ~1390 cm^−1^ are assignable to the vibration mode of the NO_3_^−^ ions and asymmetric stretch of O-NO_2_, respectively^38,40^. Similar vibration modes can be observed in the case of FT-IR spectra for LPE GS film doped with Li_2_CO_3_ and can be assigned to the vibration mode of the CO_3_^−^ ions (1340 cm^−1^) and asymmetric stretch of O-CO_2_ (~1390 cm^−1^)^41^. The same vibrational modes could be seen for LiNO_3_ and Li_2_CO_3_ standard solutions (Fig. (4b)).

From the observed FT-IR results (Fig. 4(a)) it is clear that additional peaks appear with LPE GS film chemical doping. These additional peaks match with vibrational modes of the anions in solution (Fig. 4(b)). Considering that no new peaks are visible in the given spectra (which would indicate the formation of chemical bonds) the present peaks could be a consequence of the metal salts adsorption to the graphene lattice during the doping. In order to understand Li and Au doping mechanisms XPS measurements were performed and they are presented in separate section.

### Work function modulation

**Figure 5:**
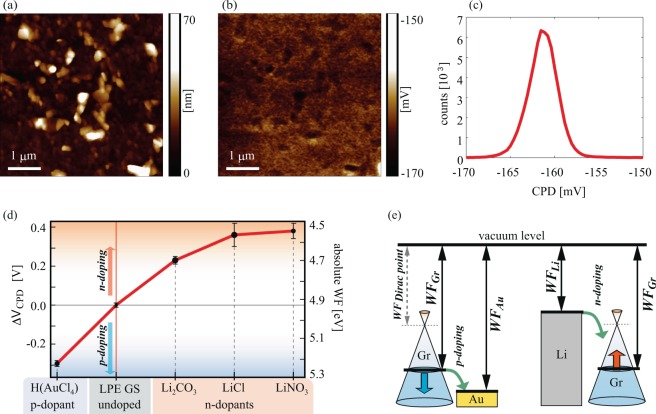
(**a**) AFM topography, (**b**) CPD map measured by KPFM, and (**c**) histogram of (**b**) shown for H(AuCl_4_) doped LPE GS film as an example. (**d**) Change in WF for doped LPE GS films for different dopants, in comparison to the undoped LPE GS film. Solid red line in (**d**) is only a guide for the eye. (**e**) Schematic representation of the work functions prior to the interaction (equal vacuum levels) for Au-based salt/graphene and Li-based salt/graphene. The green arrows indicate direction of electron flow showing that in the case of Li (Au) based salts, electrons are transferred to (from) graphene.

Results for the work function dependent on the different metal standard solution used in the LBA process are summarized in Fig. 5. The top row depicts an example with the topography (Fig. 5(a)), corresponding contact potential difference (CPD) map measured by Kelvin probe force Microscopy-KPFM (Fig. 5(b)), and the histogram of the CPD distribution measured on H(AuCl_4_) doped graphene film (Fig. 5(c)). The histogram is characterized with a single peak, which is used for the averaging and calculation of the absolute value of work function. The same procedure was done for all considered films. More details about the measurements of CPD and WF calculations are given in Supplementary information in Supplementary Figs. S3-S5. As a result, the values of the absolute work function are presented in Fig. 5(d) for both, doped and undoped LPE GS films. As can be seen, n-doping of graphene films is achieved by Li-based salts, whereas Au-based salt leads to p-doping.

The change of the WF due to the doping can be explained according to the schematic presentation in Fig. 5(e), illustrating that Li (Au) as a lower (higher) work function material compared to GS films. Therefore, presence of Li-based salts into the graphene film results in a reduction of the work function of the entire film. This behavior can be interpreted as an increase in the Fermi level of GSs – compared to the value for the undoped films – indicating predominantly a charge transfer from Li-based salts to graphene (n-doping), as expected when considering that Li has lower WF than graphene (graphite). In contrast to Li-based salts, the Au-based salt shows an opposite trend for the relative change of the work function. This indicates charge transfer from graphene to Au-based salt and a relative reduction of the Fermi level in GSs (p-doping). It is also worth mentioning that poly-crystalline nature of LPE based GS films, large amount of sheet edges and presence of the residual solvent (NMP) results in p-doped films^9^, as was also observed in the electrical measurements presented in the following subsection. Therefore, WF values are lower for the LPE-based films by at least 200 meV, than for the pristine exfoliated single-crystals^42^. p-type doping is also reflected on the WF of the reference samples (undoped LPE GS), and therefore on the whole accessible range for the WF modulation by this method. This was also highlighted in Fig. 5(e), where the *WF*_*Dirac point*_ depicts the case of undoped graphene^42^.

According to Fig. 5(d), the maximal doping in both directions is similar, around 0.3–0.4 eV, finally providing a significant range of around 0.7 eV for the work function modulation of LPE GS films. The achieved range was obtained for 0.1 mg/mL concentration of dopants. For smaller concentrations (one order of magnitude lower, 0.01 mg/mL), the observed changes in CPD were in the order of 10 mV. On the other hand, for higher concentrations (for one order of magnitude higher, 1 mg/mL) gave rise to the problems related to the formation of continuous, large-area LPE GS films, and were therefore excluded from this study. The reported shift of the Fermi level is very similar to the other (comparable) systems in the literature. WF values change of 0.3 eV in our experiment (chemical doping by Au ions) are the same order of magnitude as in Kwon *et al*. manuscripts for gold-chloride (WF change of 0.6 eV^21^, 0.6 eV^22^, 0.4 eV^25^). Compared with Kwon *et al*. alkali carbonate^23^ and chloride^25^ graphene chemical doping data (0.4 eV and 0.3–0.4 eV, respectively) WF values change for Li in our manuscript (0.2 eV and 0.4 eV) are in the same order of magnitude. Compared with literature data the same effect can be achieved but advantages of our approach is fast and simple solution-based method for one-step fabrication and WF control of large-area graphene films.

### Sheet resistance

**Figure 6:**
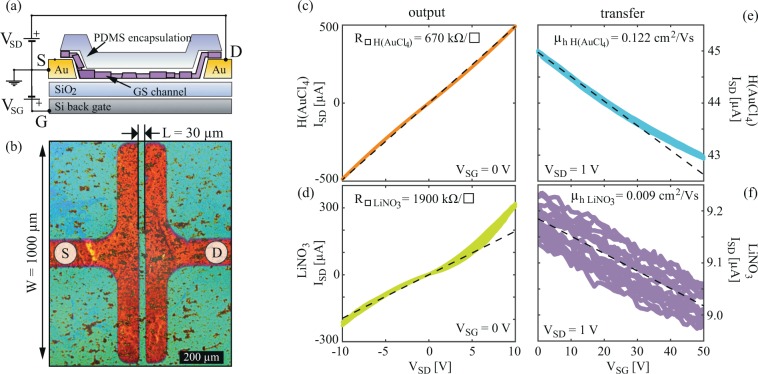
(**a**) Schematic cross-section of the bottom-contacted back-gated FET devices, also indicating electrical connections. (**b**) Optical microscopy image of one of the devices, without PDMS capping (for clarity). LBA GS film covers the entire sample surface. (**c**,**d**) Output curves of H(AuCl_4_) and LiNO_3_ doped samples, and (**e,f**) transfer curves of H(AuCl_4_) and LiNO_3_ doped samples, respectively. Dashed lines represent least squares linear fits (to selected regions) that were used to extract sheet resistance and linear mobility.

The schematic cross-section of the devices used for the electrical characterization is shown in Fig. 6(a), also indicating electrical connections. An optical microscopy image for one of the devices without PDMS encapsulation (for clarity) is shown in Fig. 6(b) illustrating source (S) and drain (D) contact geometries. One characteristic set of transport and output curves for H(AuCl_4_) and LiNO_3_ doped film is presented in Fig. 6(c–f). Here linear fits were used to extract sheet resistances and apparent linear hole mobilities. Transfer curves for all four salt-treatments and for the reference LPE GS film are presented in the Supplementary information (Supplementary Fig. S1).

In the cases of a reference (undoped) and H(AuCl)_4_ doped LPE GS samples, output curves barely deviate from a perfect linear behavior in a rather large bias range, indicating that the contact resistance is negligible in comparison to the channel. This is in contrast to all samples doped with Li-based salts, where a significant deviation from the linear output curves were observed at higher bias, indicating non-negligible contact resistance. This can be attributed to large WF differences with Au bottom contacts in the case of Li-based salt doping of the films. Furthermore, while H(AuCl_4_) doping enhances electrical performance of the films, a significant increase of the resistivity and reduction of the mobility was observed in the case of all Li-based salt dopings.

The slope of the transfer curves indicates that holes are the majority carriers for all samples, including both the undoped (reference) and all metal salt doped films. Linear fits to the transfer curves were used to estimate apparent hole mobility of the devices. While the type of majority carriers was not affected by the doping, a significant (over one order of magnitude) suppression of the field-effect was observed for Li salt dopings of the films.

**Figure 7:**
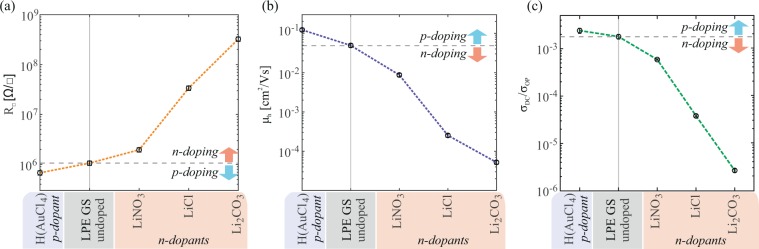
The dependence of the electrical properties of LBA graphene films on the type of metal standard solution based doping; (**a**) sheet resistance, and (**b**) apparent linear hole mobility, and (**c**) direct current conductivity to optical conductivity ratio (σ_DC_/σ_OP_). Dashed lines in (**a**–**c**) serve only as a guide for the eye.

Figure 7 summarizes electrical properties obtained for all of the measured devices as a function of the different metal based doping.

The results indicate that anions also play a significant role. In the case of Li-based salts, a large variation of the electrical properties was obtained by the different choice of the anion species. Nonetheless, the experiments point out that metal cations dictate the direction of the WF shift (see Fig. 5), as is apparent in the case of H(AuCl_4_) and LiCl where only cation species is varied. Our results of metal based doping of LPE graphene films demonstrate a tradeoff between enhancement of the electrical performance and modulation of the WF. Similar results were obtained for CVD doping with Li and Au salts^23,25^. Of a particular technological relevance is large reduction of the WF of graphene. While many methods for chemical modulation of graphene result in p-type doping^43–46^, stable and simple n-type doping is much harder to achieve^47–49^. For an efficient electron injection, a significant reduction of graphene’s WF is required. As pointed out by WF measurements and electrical characterization, LiNO_3_ is the best choice from the tested Li-based salts with respect to both the largest WF reduction (by 400 meV) and least deterioration of the electrical properties of the films with ~2–3 times increase in sheet resistance compared to the reference (undoped LPE GS).

In contrast, doping of LPE GS films by HNO_3_ vapor results in an increase of the apparent mobility^9^. However, using a LiNO_3_ solution reduces the mobility by one order of magnitude. Therefore, Li^+^ cations – and not anions – are likely responsible for the deterioration of the electrical properties upon n-doping. An increase of sheet resistance was observed in doping of CVD graphene with alkali metal carbonates and chlorides^23,25^. There, a significant increase in the sheet resistance was related to the combination of carbon atoms and dopant metals because electron donation occurred^23,25^. Also, Chen *et al*. observed that the mobility of the charge carriers decreases with the increase of the potassium doping concentration which they attributed to additional scattering caused by ionized potassium atoms^49,50^. It is most likely that Li^+^ cations are acting as scattering centers for the carriers, or provide traps at the boundaries between neighbouring GSs and effectively increase contact resistance between the overlapping GSs.

Finally, considering that the main potential application of these LPE GS films lies in transparent electrodes, direct current conductivity to optical conductivity ratio (σ_DC_/σ_OP_) is presented in Fig. 7(c) for all metal standard solution doping cases and for the reference (undoped). σ_DC_/σ_OP_ is a parameter frequently reported in order to characterize the relative performance of the films in terms of transparency and sheet resistance^11,33,51^. The higher the ratio the better the quality of transparent electrodes^33^. Compared to the changes in the electrical properties (Fig. 7(a)) the changes in the optical properties (Fig. 2) are minor. Therefore, the dependence of the σ_DC_/σ_OP_ on the type of the metal-ion doping clearly follows the trend set by 1/R_□_.

### X-ray Photoemission Spectroscopy (XPS) measurements

**Figure 8:**
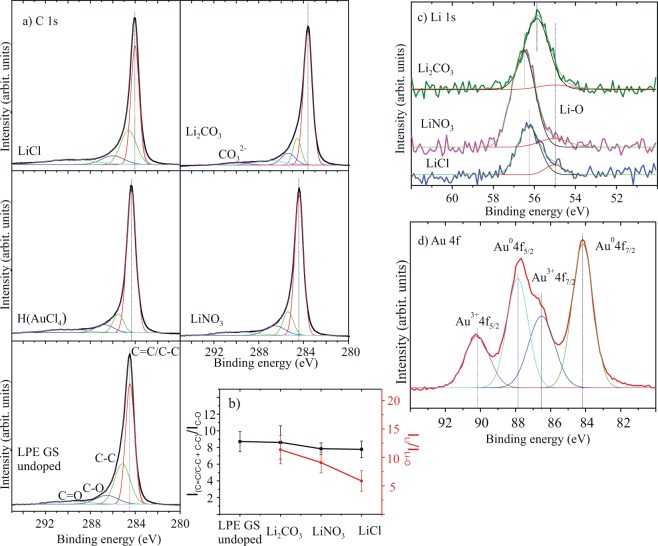
(**a**) XPS C 1 s spectra of undoped and H(AuCl_4_), LiCl, LiNO_3_, Li_2_CO_3_ doped LPE GS films. C=C/C–C in aromatic rings (284.5 eV); C–C sp^3^ (285.4 eV); C–O (286.6 eV) and C=O (289 eV) were considered. For Li_2_CO_3_ a small additional peak at 289.2–291.0 eV can be assigned to carbonate. (**b**) Peak intensity ratio for the sum of C=C/C-C and C-C peaks intensities, and the intensity of C-O, I_(C=C/C-C+C-C)_/I_(C-O)_ (black line) and the ratio of Li 1 s intensity from Li salts to Li-O intensity, I_Li_/I_Li-O_ (red line). (**c**) XPS Li 1 s spectra for different Li compounds and for Li–O. (**d**) The Au 4 f peak in the XPS data of H(AuCl_4_).

In order to understand Au and Li ion doping mechanisms XPS measurements were performed. C 1 s, Au 4 f and Li 1 s core-level XPS spectra are shown in Fig. 8. N 1 s, Cl 2p and O 1 s spectra are presented as Supplementary Fig. S2. The C 1 s peak of undoped and LiCl, LiNO_3_, Li_2_CO_3_, H(AuCl_4_) doped LPE GS films is shown on Fig. 8(a). The C 1 s peak is deconvoluted using Gaussian profile into 4 components for undoped and doped films: C=C/C–C in aromatic rings (284.5 eV); C–C sp^3^ (285.4 eV); C–O (286.6 eV) and C=O (289 eV)^23,52^. In the case of Li_2_CO_3_ we can see a small additional peak at 289.2–291.0 eV^53^ which can be assigned to carbonate. Detected oxygen peak (C=O) is likely due to the residual of NMP and oxygen functionalized edges (C–O) on graphene^54,55^. The C=C/C–C peak was shifted to a lower binding energy by about 0.16, 0.48, 0.10 and 0.83 eV for H(AuCl_4_), LiCl, LiNO_3_ and Li_2_CO_3_ doping process, respectively. The C=C/C–C peak shifts in present work are a consequence of doping by different metal standard solutions. Kwon *et al*. have shown that degree of doping was related to the electronegativity of the anion in the Au complex where anions with a high electronegativity and high bond strength are adequate for use as a p-type dopant in graphene^21^. Thus, different shifts of C=C/C–C peak for different metal-salt doping materials could be also a consequence of anions influence on graphene films.

Figure 8(c) show the Li 1 s core-level XPS spectra. Literature values for Li 1 s core-level for different Li compounds are: LiCl (56.2 eV), Li_2_CO_3_ (55.5 eV) and LiNO_3_ (55.8 eV)^56^ and they correspond well to the values obtained in this work. Li 1 s peak at 55.0 eV is assigned to Li–O bond^57^. Vijayakumar and Jianzhi have shown that lithium ion tends to bind with the oxygen rather than the carbon on graphene surface, and interacts by forming Li-O ionic bond^58^. Also Kwon *et al*. have proposed that C–O–X complexes can be formed during doping treatment and can act as an additional dipole to further reduce the value of WF^23–25,59^. The intensity ratio between sum of the intensities of C=C/C-C and C-C peaks, and the intensity of C-O (I_(C=C/C-C+C-C)_/I_(C-O)_) is shown in Fig. 8(b). Also, the ratio of Li 1 s intensity from Li salts to Li-O intensity (I_Li_/I_Li-O_) can be seen in Fig. 8(b). In both cases, intensity ratios decrease for Li_2_CO_3_, LiNO_3_, LiCl, respectively and this implies increased formation of C–O and Li–O bonds. Increased number of Li-O bonds follow the increasing trend of C–O bonds, which is in correlation with the WF change (Fig. (5d)). The above mentioned results strongly suggest that the mechanism of n-type doped LPE GS films with lithium-salts could be explained with formation of Li complexes (C–O–Li).

Figure 8(d) show the Au 4 f peak of gold-chloride doped LPE GS film. The peak is composed of metal (Au^0^) and metal ion (Au^3+^). The peaks at 84.2 eV and 87.9 eV are assigned to neutral Au (Au^0^ 4f_7/2_ and Au^0^ 4f_5/2_, respectively), and the peaks at 86.5 eV and 90.2 eV are assigned to Au ion (Au^3+^ 4f_7/2_ and Au^3+^ 4f_5/2_, respectively). Au ions (Au^3+^) have positive reduction potential and have tendency to spontaneously accept charges from other materials (graphene) and reduce to Au^0^ ^21,22,25,60^. Therefore, the mechanism of p-doped LPE GS film can be explained as spontaneous electron transfer from graphene film to Au^3+^, resulting in depletion of electrons in the graphene networks, thus increasing the WF of doped graphene.

## Conclusion

We demonstrate a straightforward single-step method for forming and doping of LPE GS films by metal standard solutions through charge transfer processes. Chemical doping of graphene allows to modulate its WF in a very large range, and therefore potentially enables to use the same electrode material for both, the injection and for the extraction of the electrons. n-doping of graphene films is achieved by Li-based salts, whereas Au-based salt leads to p-doping. Furthermore, solution-processed graphene films are in particular suited for the chemical modulations, since a large number of the sheet edges opens up many adsorption sites and enhances the doping effects when compared to many other types of graphene.

The morphology of the LPE GS films does not change with the doping process, except that doped films contain agglomerates. FT-IR measurements point out that graphene basal planes stay chemically unchanged with metal doping and the charge transfer process is enabled with adsorption of the metal salts. Li-based salts decrease the WF, while Au-based salts increase the WF of the entire film. The maximal doping in both directions gives a significant range of around 0.7 eV for the work function modulation. Changing the dopant (Au or Li based salts) significantly affects the electrical properties of the films. In the case of the Li-based salts doping of the film, a significant suppression of the field-effect mobility and the increase of the sheet resistance was observed. This indicates that adsorbed Li-anions act as scattering centers for the charges. XPS data indicated that different mechanisms exist in the case of Au and Li doping. For Au ions spontaneous charge transfer occurred from graphene, thus increasing WF. In the case of Li doping, potential adsorption sites are a large number of the sheet edges where C-O bonds are preferential sites for lithium ions and for forming of C-O-Li complexes. In all cases graphene films are p-type, which is in accordance with KPFM measurements. Also, tradeoff between Li complex which reduce the value of WF and anion which increase the value of WF could be a reason of such a doping.

Metal salts charge transfer doping – which happens with this single-step method – provides a facile and effective method to tune the WF of LPE graphene therefore extending the potential use of these materials in low-cost transparent electrode applications.

## Methods

### Preparation of GS dispersion and doping solutions

A dispersion of GS in N-methyl-2-pyrrolidone (NMP, Sigma Aldrich, product no. 328634) has been used. GS dispersion was prepared from graphite powder (Sigma Aldrich, product no. 332461) of initial concentration 18 mg/mL. The solution was sonicated in a low-power ultrasonic bath for 14 h. The resulting dispersion was centrifuged for 60 min at 3000 rpm immediately after the sonication.

Stock standard solutions used in our work for n-doping are 1 mg/mL LiCl, LiNO_3_ and Li_2_CO_3_ and for p-doping is 1 mg/mL gold standard solution (Merck, H(AuCl_4_), product no. 170216). Lithium standard solutions were prepared from originated Li salts (LiCl, LiNO_3_ and Li_2_CO_3_, Merck, product no. 105679, 105653 and 105680, respectively). By appropriate dilution of the stock solution with deionized water we obtained 0.1 mg/mL metal water solution which is then used in doping process.

### Deposition on a substrate and doping of LPE GS films

GS dispersion in NMP was used to fabricate transparent and conductive films by LBA technique at a water-air interface, like in our previous work^9,29,61^. A small amount of GS dispersion was added to the water-air interface and after the film was formed it was slowly scooped onto the target substrate. Applying the same process of fabricating the GS films and using the appropriate metal standard solution instead of water, chemical doping was achieved. As substrates SiO_2_/Si wafer were used for electrical and WF measurements, while quartz and CaF_2_ substrates were chosen for optical and FT-IR spectroscopy, respectively.

### Characterization of undoped and doped LPE GS films

The Morphology of LPE GS films was studied by optical and atomic force microscopy (AFM). Topographic AFM measurements were done by NTEGRA Prima AFM system and NSG01 probes with a typical tip radius of around 10 nm. The surface roughness of LPE GS films was calculated as a root-mean square of the height distribution and averaged on ten 50 × 50 μm^2^ areas.

Kelvin probe force microscopy (KPFM) – established almost three decades ago^62^ and in the meantime frequently applied to graphene^42,63–65^ – was employed in order to characterize changes in the electrical surface potential and corresponding Fermi level shifts due to doping. For this purpose, we measured the contact potential difference (CPD) between AFM tip and the sample surface^66^ by using Pt covered NSG01/Pt probes with a typical tip curvature radius of 35 nm. In the first pass of KPFM, the sample topography was measured in tapping AFM mode. In the second pass, the probe was lifted by 20 nm, and moved along the trajectory measured in the first pass. Simultaneously, the sum of AC and DC voltage was applied between the sample and the probe. The AC voltage excites AFM probe oscillations during its movement, while the CPD between AFM tip and the sample surface in every point is then equal to the value of variable DC voltage which cancels the AFM probe oscillations. For all samples, the CPD was measured on five 5 × 5 μm^2^ areas, and then averaged. In order to obtain the absolute value of the work function, the following procedure was applied^42^. The CPD is equal to the work function difference between AFM tip (WF_t_) and sample (WF_s_), CPD = WF_t_-WF_s_. The calibration of the WF_t_ was done by a standard procedure consisting of KPFM measurements on a freshly cleaved HOPG with a well known work function of 4.6 eV^42^. Finally, the sample work function was calculated as WF_s_ = WF_t_ - CPD, where CPD is measured by KPFM for all, undoped and doped LPE GS films.

The effect of chemical doping on optical properties of LBA GS films was investigated with measurements of optical transmittance, using UV-VIS spectrophotometer (Beckman Coulter DU 720 UV-VIS Spectrophotometer).

Electrical measurements were performed under ambient conditions in a standard field-effect device configuration with Si substrate acting as a back gate electrode, using Keithley 2636 A SYSTEM SourceMeter. Devices were based on bottom-contact gold pads defined by a shadow mask with L/W = 30 µm/1000 µm, and SiO_2_ as a gate dielectric with thickness of 285 nm. Graphene films were deposited using the same LBA method as described above. The top surface of the devices was encapsulated by polydimethylsiloxane (PDMS) films (GelPak X4) to ensure stable performance and minimize any adsorption/desorption during electrical measurements that could occur from the surroundings (e.g. water vapor). Electrical characterization was performed on several devices of each doping with metal standard solution, and for undoped films as a reference. For each device ten subsequent forward and backward transfer and output curves were measured, using low sweeping rate (~0.005–1 Hz per point in a voltage sweep) to minimize parasitic capacitance. Sheet resistance and apparent linear field-effect mobility were extracted using fits to output and transfer curves, respectively. For the output measurements source-drain bias was varied in a range between −10 V and +10 V, with the gate electrode grounded. For transfer measurements, the gate voltage was varied between 0 V and 50 V, with source-drain bias at 1 V in all cases except for Li_2_CO_3_ where due to a very weak field-effect (very low mobility) 10 V bias was used.

The room-temperature micro-Raman spectra of undoped and metal salt doped LPE GS films were collected using Tri Vista 557 triple spectrometer coupled to the liquid nitrogen-cooled CCD detector. Nd:YAG laser line of 532 nm was used for the excitation and 50 magnification objective was used for focusing the beam onto the sample. Low laser power (less than 1 mW) was applied to prevent the thermal degradation of the sample. Each LPE GS film sample was measured at eight different positions.

Fourier transform infrared absorbance spectra (FT-IR spectra) of undoped and metal salt doped LPE GS films were measured over a range of 400–4000 cm^−1^ with Nicolet Nexus 470 FT-IR spectrometer. Standard solutions which were used for the preparation of doped films were measured too and they were prepared by drop casting method on the CaF_2_ substrate.

XPS spectra were recorded using a Thermo Scientific instrument (K-Alpha spectrometer, Thermo Fisher Scientific, Waltham, USA) equipped with a monochromatic Al Kα X-ray source (1486.6 eV). High-resolution scans were performed with a pass energy of 50 eV and a step size of 0.1 eV. All analyses were performed at room temperature.

## Supplementary information

Supplementary Information.

Supplementary Information2.

## Data Availability

The datasets obtained and analysed during the current study that are not included in this article are available from the corresponding authors on reasonable request.

## References

[1] Geim AK (2009). Graphene: Status and prospects. Science.

[2] Geim AK, Novoselov KS (2007). The rise of graphene. Nat. Mater..

[3] Novoselov KS (2007). Room-temperature quantum hall effect in graphene. Science.

[4] Bonaccorso F, Sun Z, Hasan T, Ferrari AC (2010). Graphene photonics and optoelectronics. Nat. Photonics.

[5] Kwon KC, Kim BJ, Lee JL, Kim SY (2013). Role of ionic chlorine in the thermal degradation of metal chloride-doped graphene sheets. J. Mater. Chem. C.

[6] Ferrari AC (2015). Science and technology roadmap for graphene, related two-dimensional crystals, and hybrid systems. Nanoscale.

[7] Backes C (2017). Guidelines for exfoliation, characterization and processing of layered materials produced by liquid exfoliation. Chem. Mater..

[8] Li X (2008). Highly conducting graphene sheets and Langmuir-Blodgett films. Nat. Nanotechnol..

[9] Matković A (2016). Enhanced sheet conductivity of Langmuir-Blodgett assembled graphene thin films by chemical doping. 2D Mater.

[10] Lee SK (2012). All graphene-based thin film transistors on flexible plastic substrates. Nano Lett..

[11] Zheng Q (2011). Transparent Conductive Films Consisting of Ultralarge Graphene Sheets Produced by Langmuir-Blodgett Assembly. ACS Nano.

[12] Park J (2011). Work-function engineering of graphene electrodes by self-assembled monolayers for high-performance organic field-effect transistors. J. Phys. Chem. Lett..

[13] Tong SW, Wang Y, Zheng Y, Ng MF, Loh KP (2011). Graphene intermediate layer in tandem organic photovoltaic cells. Adv. Funct. Mater..

[14] Wang Y, Tong SW, Xu XF, Özyilmaz B, Loh KP (2011). Interface engineering of layer-by-layer stacked graphene anodes for high-performance organic solar cells. Adv. Mater..

[15] Wu J (2010). Organic light-emitting diodes on solution-processed graphene transparent electrodes. ACS Nano.

[16] Wang X, Zhi L, Mullen K (2008). Transparent, conductive graphene electrodes for dye-sensitized solar cells. Nano Lett..

[17] Alfano B (2016). Modulating the sensing properties of graphene through an eco-friendly metal-decoration process. Sensors Actuators, B Chem..

[18] Lynch P, Khan U, Harvey A, Ahmed I, Coleman JN (2016). Graphene-MoS2 nanosheet composites as electrodes for dye sensitised solar cells. Mater. Res. Express.

[19] Mosciatti T (2015). A multifunctional polymer-graphene thin-film transistor with tunable transport regimes. ACS Nano.

[20] Giubileo F, Di Bartolomeo A (2017). The role of contact resistance in graphene field-effect devices. Prog. Surf. Sci..

[21] Kwon KC, Kim BJ, Lee JL, Kim SY (2013). Effect of anions in Au complexes on doping and degradation of graphene. J. Mater. Chem. C.

[22] Kwon KC, Choi KS, Kim SY (2012). Increased work function in few-layer graphene sheets via metal chloride doping. Adv. Funct. Mater..

[23] Kwon KC, Choi KS, Kim BJ, Lee JL, Kim SY (2012). Work-function decrease of graphene sheet using alkali metal carbonates. J. Phys. Chem. C.

[24] Huang JH, Fang JH, Liu CC, Chu CW (2011). Effective work function modulation of graphene/carbon nanotube composite films as transparent cathodes for organic optoelectronics. ACS Nano.

[25] Kwon KC, Choi KS, Kim C, Kim SY (2014). Role of metal cations in alkali metal chloride doped graphene. J. Phys. Chem. C.

[26] Wang X, Xu JB, Xie W, Du J (2011). Quantitative analysis of graphene doping by organic molecular charge transfer. J. Phys. Chem. C.

[27] Shin HJ (2010). Control of electronic structure of graphene by various dopants and their effects on a nanogenerator. J. Am. Chem. Soc..

[28] Shi Y (2010). Work function engineering of graphene electrode via chemical doping. ACS Nano.

[29] Tomašević-Ilić T (2018). Reducing sheet resistance of self-assembled transparent graphene films by defect patching and doping with UV/ozone treatment. Appl. Surf. Sci..

[30] Matković A (2012). Spectroscopic imaging ellipsometry and Fano resonance modeling of graphene. J. Appl. Phys..

[31] Bracamonte MV, Lacconi GI, Urreta SE, Foa Torres LEFF (2014). On the nature of defects in liquid-phase exfoliated graphene. J. Phys. Chem. C.

[32] Eckmann A (2012). Probing the nature of defects in graphene by Raman spectroscopy. Nano Lett..

[33] Rytel K (2018). Ultrasonication-induced sp3 hybridization defects in Langmuir-Schaefer layers of turbostratic graphene. Phys. Chem. Chem. Phys..

[34] Eckmann A, Felten A, Verzhbitskiy I, Davey R, Casiraghi C (2013). Raman study on defective graphene: Effect of the excitation energy, type, and amount of defects. Phys. Rev. B - Condens. Matter Mater. Phys..

[35] Drewniak S (2016). Studies of reduced graphene oxide and graphite oxide in the aspect of their possible application in gas sensors. Sensors.

[36] Kim WJ, Basavaraja C, Thinh PX, Huh DS (2013). Structural characterization and DC conductivity of honeycomb-patterned poly(ε-caprolactone)/gold nanoparticle-reduced graphite oxide composite films. Mater. Lett..

[37] Ţucureanu V, Matei A, Avram AM (2016). FTIR spectroscopy for carbon family study. Crit. Rev. Anal. Chem..

[38] Wu X (2015). One-step freezing temperature crystallization of layered rare-earth hydroxide (Ln2(OH)5NO3·nH2O) nanosheets for a wide spectrum of Ln (Ln = Pr-Er, and Y), anion exchange with fluorine and sulfate, and microscopic coordination probed via photoluminescence. J. Mater. Chem. C.

[39] Nakamoto, K. Infrared and Raman Spectra of Inorganic and Coordination Compounds. Part A: Theory and Applications in Inorganic Chemistry; Part B: Application in Coordination, Organometallic, and Bioinorganic Chemistry, 5th Edition (Nakamoto, Kazuo). *John Wiley and Sons* (John Wiley and Sons, 2009).

[40] Geng F (2008). New layered rare-earth hydroxides with anion-exchange properties. Chem. Eur. J..

[41] Lefèvre G (2004). *In situ* Fourier-transform infrared spectroscopy studies of inorganic ions adsorption on metal oxides and hydroxides. Adv. Colloid Interface Sci..

[42] Yu Y (2009). Tuning the graphene work function by electric field effect. Nano Lett..

[43] Levesque PL (2011). Probing charge transfer at surfaces using graphene transistors. Nano Lett..

[44] Kuruvila A (2014). Organic light emitting diodes with environmentally and thermally stable doped graphene electrodes. J. Mater. Chem. C.

[45] Meyer J (2014). Metal oxide induced charge transfer doping and band alignment of graphene electrodes for efficient organic light emitting diodes. Sci. Rep.

[46] Matković A (2017). Probing charge transfer between molecular semiconductors and graphene. Sci. Rep.

[47] Sanders S (2015). Engineering high charge transfer n-doping of graphene electrodes and its application to organic electronics. Nanoscale.

[48] Han KS (2016). A non-destructive n-doping method for graphene with precise control of electronic properties via atomic layer deposition. Nanoscale.

[49] Chen JH (2008). Charged-impurity scattering in graphene. Nat. Phys..

[50] Pinto H, Markevich A (2014). Electronic and electrochemical doping of graphene by surface adsorbates. Beilstein J. Nanotechnol.

[51] De S, Coleman JN (2010). Are there fundamental limitations on the sheet resistance and transmittance of thin graphene films?. ACS Nano.

[52] Benayad A (2009). Controlling work function of reduced graphite oxide with Au-ion concentration. Chem. Phys. Lett..

[53] López GP, Castner DG, Ratner BD (1991). XPS O 1s binding energies for polymers containing hydroxyl, ether, ketone and ester groups. Surf. Interface Anal..

[54] Hernandez Y (2008). High-yield production of graphene by liquid-phase exfoliation of graphite. Nat. Nanotechnol.

[55] Kim H (2013). Optoelectronic properties of graphene thin films deposited by a Langmuir-Blodgett assembly. Nanoscale.

[56] Naumkin, A. V., Kraut-Vass, A., Gaarenstroom, S. W. & Powell, C. J. NIST X-ray photoelectron spectroscopy database. Available at: https://srdata.nist.gov/xps/EngElmSrchQuery.aspx?EType=PE&CSOpt=Retri_ex_dat&Elm=Li. (2019).

[57] Moulder, J. F., Stickle, W. F., Sobol, P. E. & Bomben, K. D. *Hanbook of X-ray photoelectron spectroscopy*. *Reference book of standard spectra for indentification and interpretation of XPS data* (Perkin-Elmer Corporation, Physical Electronic division, 1992).

[58] Vijayakumar M, Jianzhi H (2013). Exploring the interaction between lithium ion and defective graphene surface using dispersion corrected DFT studies. ECS Trans.

[59] Pickett WE (1994). Negative electron affinity and low work function surface: Cesium on oxygenated diamond (100). Phys. Rev. Lett..

[60] Syu JY (2016). Wide-range work-function tuning of active graphene transparent electrodes via hole doping. RSC Adv..

[61] Tomašević-Ilić T (2016). Transparent and conductive films from liquid phase exfoliated graphene. Opt. Quantum Electron..

[62] Nonnenmacher M, O’Boyle MP, Wickramasinghe HK (1991). Kelvin probe force microscopy. Appl. Phys. Lett..

[63] Vasić B (2013). Atomic force microscopy based manipulation of graphene using dynamic plowing lithography. Nanotechnology.

[64] Vasić, B. *et al*. Low-friction, wear-resistant, and electrically homogeneous multilayer graphene grown by chemical vapor deposition on molybdenum. *Appl. Surf. Sci*. **509**, 144792 (2020).

[65] Panchal V, Pearce R, Yakimova R, Tzalenchuk A, Kazakova O (2013). Standardization of surface potential measurements of graphene domains. Sci. Rep.

[66] Udum Y (2014). Inverted bulk-heterojunction solar cell with cross-linked hole-blocking layer. Org. Electron..

