# Supplementary material for: Single-step fabrication and work function engineering of Langmuir-Blodgett assembled few-layer graphene films with Li and Au salts
Source: Sci Rep. 2020 May 21;10:8476. doi: 10.1038/s41598-020-65379-1 (PMC7242397; doi:10.1038/s41598-020-65379-1)
Supplement: Supplementary file 1 — Supplementary Information. [file 41598_2020_65379_MOESM1_ESM.pdf]

Single-step fabrication and work function engineering of Langmuir-Blodgett assembled few-layer graphene films with Li and Au salts

Ivana R. Milošević[1,*], Borislav Vasić[1], Aleksandar Matković[2,*], Jasna Vujin[1], Sonja Aškrabić[3], Markus Kratzer[2], Thomas Griesser[4], Christian Teichert[2] and Radoš Gajić[1]

[1]Laboratory for Graphene, other 2D Materials and Ordered Nanostructures of Center for Solid State Physics and New Materials, Institute of Physics, University of Belgrade, Pregrevica 118, 11080 Belgrade, Serbia
[2]Institute of Physics, Montanuniversität Leoben, Franz Josef Str. 18, 8700 Leoben, Austria
[3]Nanostructured Matter Laboratory of Center for Solid State Physics and New Materials, Institute of Physics, University of Belgrade, Pregrevica 118, 11080 Belgrade, Serbia
[4]Institute of Chemistry of Polymeric Materials, Montanuniversitaet Leoben, Otto-Gloeckel-Straße 2, 8700 Leoben, Austria

[*]novovic@ipb.ac.rs
[*]aleksandar.matkovic@unileoben.ac.at

S1: Transfer characteristics

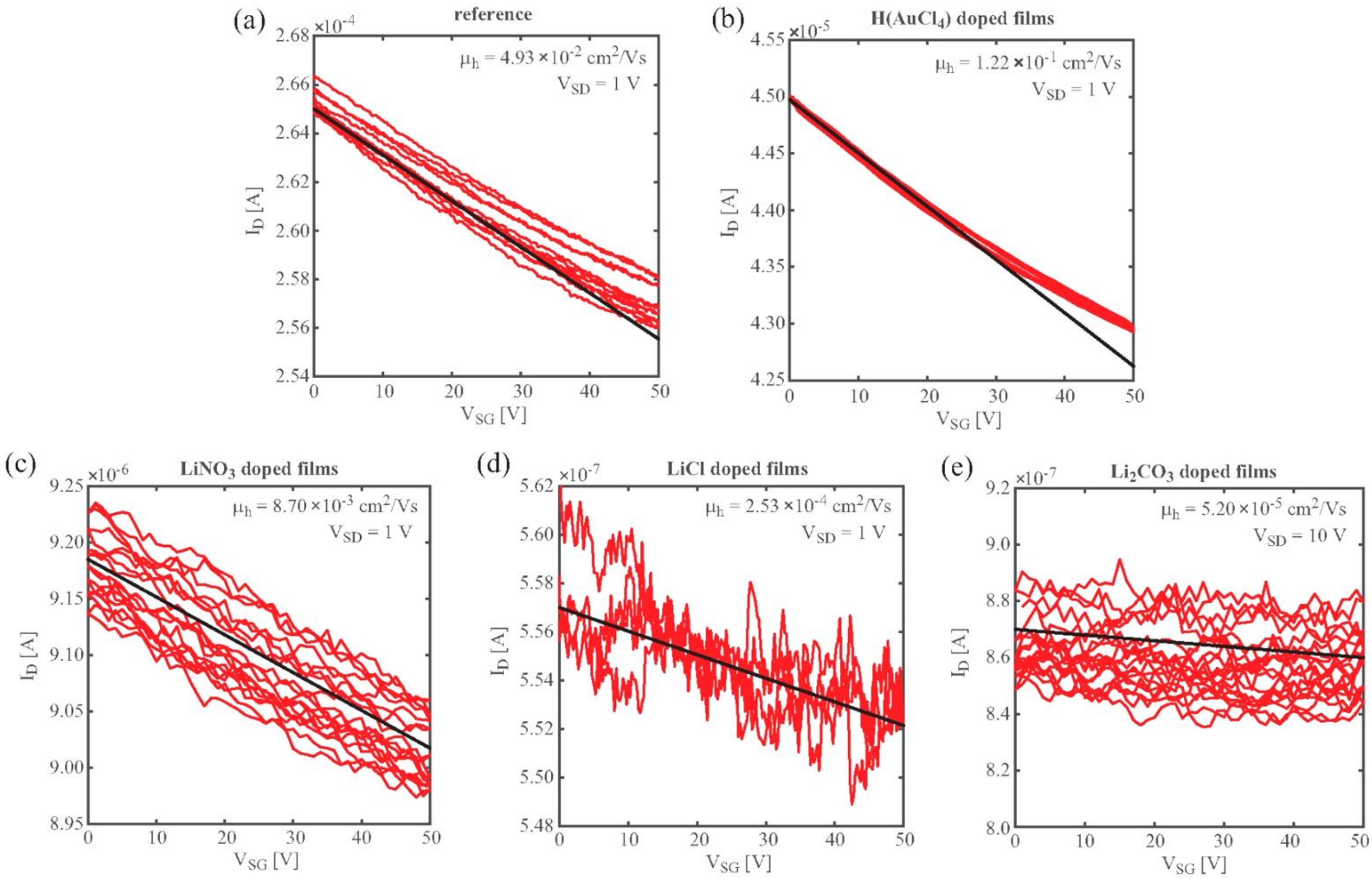

Figure S1: Transfer characteristics. (a) Reference LBA GS film, formed at the interface DI water-air without introduction of salts. (b-e) $H(AuCl_4)$, $LiNO_3$, LiCl, and Li_2CO_3 doped LBA GS films. Red lines represent five subsequent forward and backward sweeps. Black lines are linear fits used to estimate apparent hole mobility of the FETs. Source-drain bias (V_{SD}) was set to 1 V in all cases, except for Li_2CO_3-doping where due to low conductivity of the films V_{SD} = 10 V. The slopes of the curves indicate that within the accessible range of source-gate bias (V_{SG}), holes are majority carriers. Charge neutrality point was not reached within the available V_{SG} range (limited by SiO_2 dielectric breakdown). The gate leakage current was in all cases below 1 nA.

S2: X-ray Photoemission Spectroscopy core-level spectra for O 1s, N 1s and Cl 2p

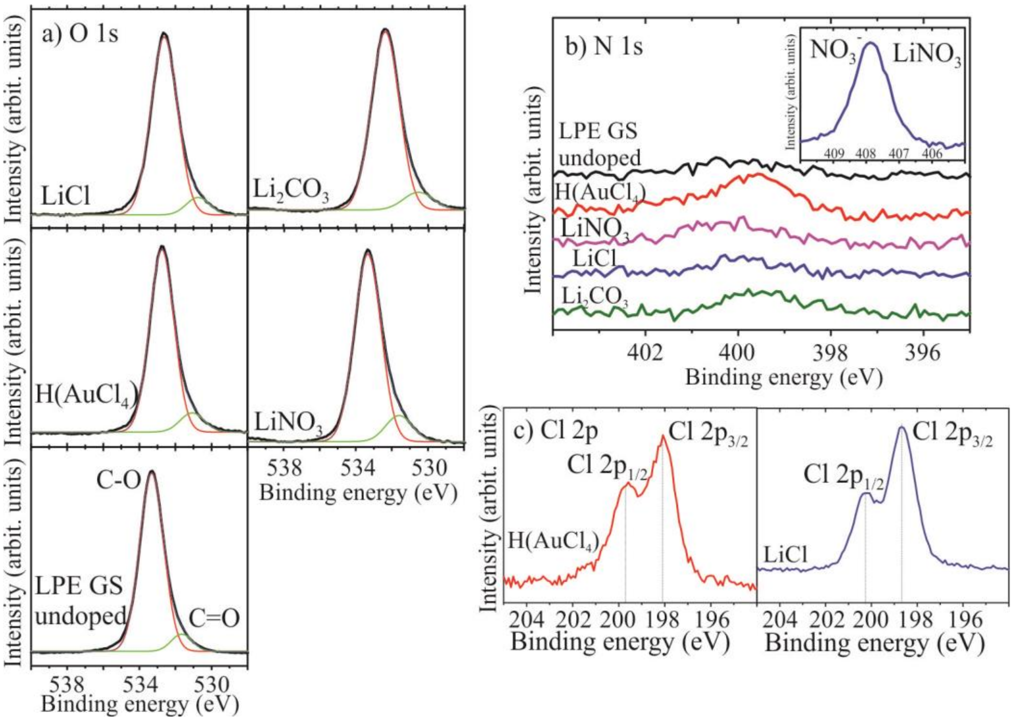

Figure S2: (a) The O 1s core-level XPS spectra for LPE GS undoped and H($AuCl_4$), $LiNO_3$, LiCl and Li_2CO_3 doped films. The O 1s spectra can be deconvoluted in 2 components: C-O (533.6 eV) and C=O (532 eV)[1]. (b) The XPS N 1s spectra (400.0 eV) indicating nitrogen[2,3] presence in undoped and metal-doped films likely due to the residual of NMP/Nitrate peak[4] for $LiNO_3$ doped graphene films at 407.3 eV (inset). c) The XPS Cl 2p spectra for H($AuCl_4$) and LiCl doped LBA GS films. Literature data for Cl $2p_{3/2}$ and Cl $2p_{1/2}$ in the case of H($AuCl_4$) are 198.4 eV[4] and 199.8 eV[5] and for LiCl are 198.8 eV[4] and 200.4[4] eV, respectively.

1. Matković, A. *et al.* Enhanced sheet conductivity of Langmuir-Blodgett assembled graphene thin films by chemical doping. *2D Mater.* **3**, 015002 (2016).
2. Sun, H. *et al.* Binder-free graphene as an advanced anode for lithium batteries. *J. Mater. Chem. A* **4**, 6886–6895 (2016).
3. Thodkar, K. *et al.* Restoring the Electrical Properties of CVD Graphene via Physisorption of Molecular Adsorbates. *ACS Appl. Mater. Interfaces* **9**, 25014–25022 (2017).
4. Naumkin, A. V., Kraut-Vass, A., Gaarenstroom, S. W. & Powell, C. J. NIST X-ray photoelectron spectroscopy database. Available at: https://srdata.nist.gov/xps/EngElmSrchQuery.aspx?EType=PE&CSOpt=Retri_ex_dat&Elm=Li (2019).
5. Syu, J. Y. *et al.* Wide-range work-function tuning of active graphene transparent electrodes via hole doping. *RSC Adv.* **6**, 32746–32756 (2016).
