# Supplementary material for: Single-step fabrication and work function engineering of Langmuir-Blodgett assembled few-layer graphene films with Li and Au salts
Source: Sci Rep. 2020 May 21;10:8476. doi: 10.1038/s41598-020-65379-1 (PMC7242397; doi:10.1038/s41598-020-65379-1)
Supplement: Supplementary file 2 — Supplementary Information2. [file 41598_2020_65379_MOESM2_ESM.pdf]

Single-step fabrication and work function engineering of Langmuir-Blodgett assembled few-layer graphene films with Li and Au salts

Ivana R. Milošević[1,*], Borislav Vasić[1], Aleksandar Matković[2,*], Jasna Vujin[1], Sonja Aškrabić[3], Markus Kratzer[2], Thomas Griesser[4], Christian Teichert[2] and Radoš Gajić[1]

[1]Laboratory for Graphene, other 2D Materials and Ordered Nanostructures of Center for Solid State Physics and New Materials, Institute of Physics, University of Belgrade, Pregrevica 118, 11080 Belgrade, Serbia
[2]Institute of Physics, Montanuniversität Leoben, Franz Josef Str. 18, 8700 Leoben, Austria
[3]Nanostructured Matter Laboratory of Center for Solid State Physics and New Materials, Institute of Physics, University of Belgrade, Pregrevica 118, 11080 Belgrade, Serbia
[4]Institute of Chemistry of Polymeric Materials, Montanuniversitaet Leoben, Otto-Gloeckel-Straße 2, 8700 Leoben, Austria

[*]novovic@ipb.ac.rs
[*]aleksandar.matkovic@unileoben.ac.at

S1: Transfer characteristics

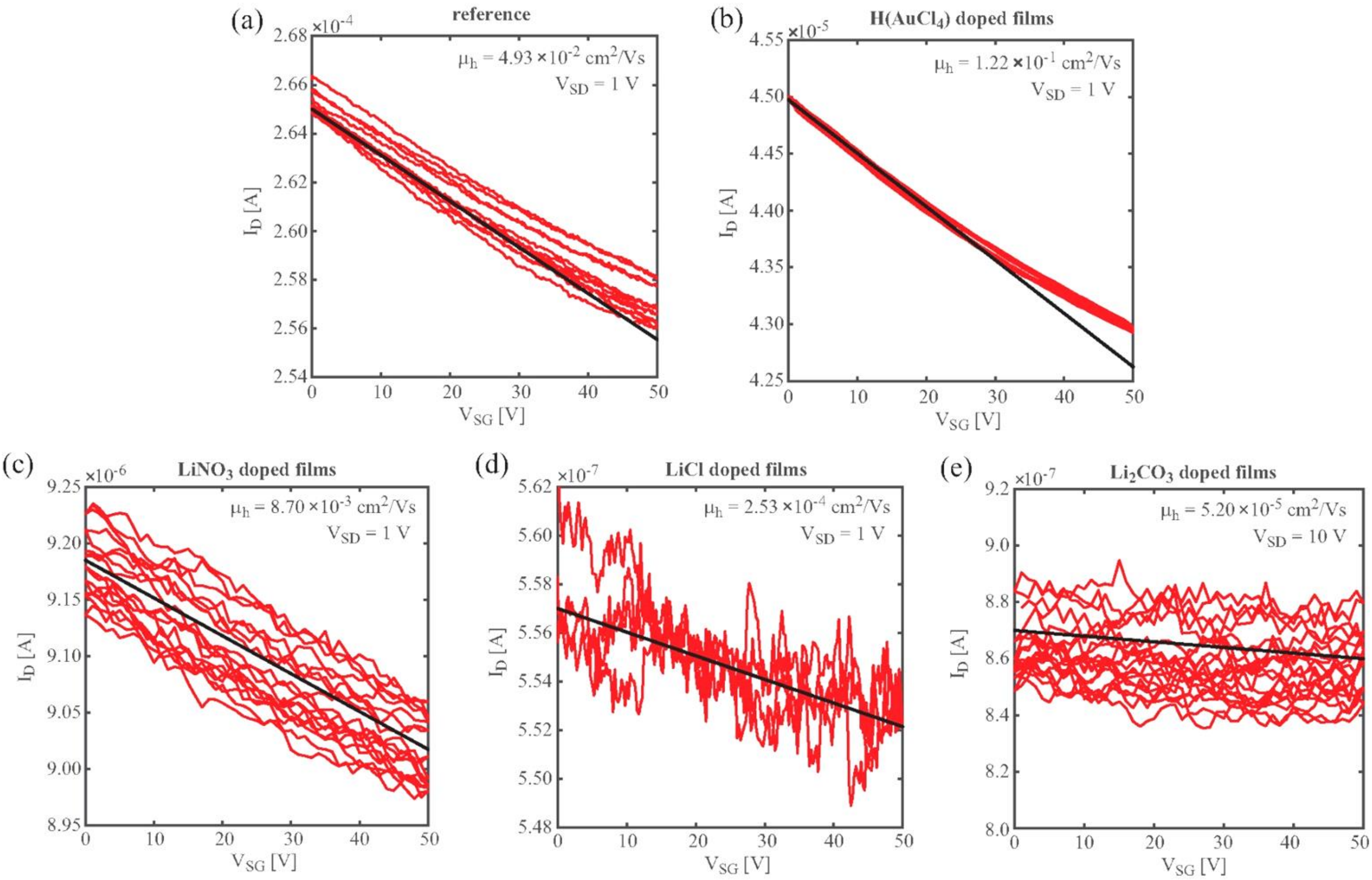

Figure S1: Transfer characteristics. (a) Reference LBA GS film, formed at the interface DI water-air without introduction of salts. (b-e) $H(AuCl_4)$, $LiNO_3$, LiCl, and Li_2CO_3 doped LBA GS films. Red lines represent five subsequent forward and backward sweeps. Black lines are linear fits used to estimate apparent hole mobility of the FETs. Source-drain bias (V_{SD}) was set to 1 V in all cases, except for Li_2CO_3-doping where due to low conductivity of the films V_{SD} = 10 V. The slopes of the curves indicate that within the accessible range of source-gate bias (V_{SG}), holes are majority carriers. Charge neutrality point was not reached within the available V_{SG} range (limited by SiO_2 dielectric breakdown). The gate leakage current was in all cases below 1 nA.

S2: X-ray Photoemission Spectroscopy core-level spectra for O 1s, N 1s and Cl 2p

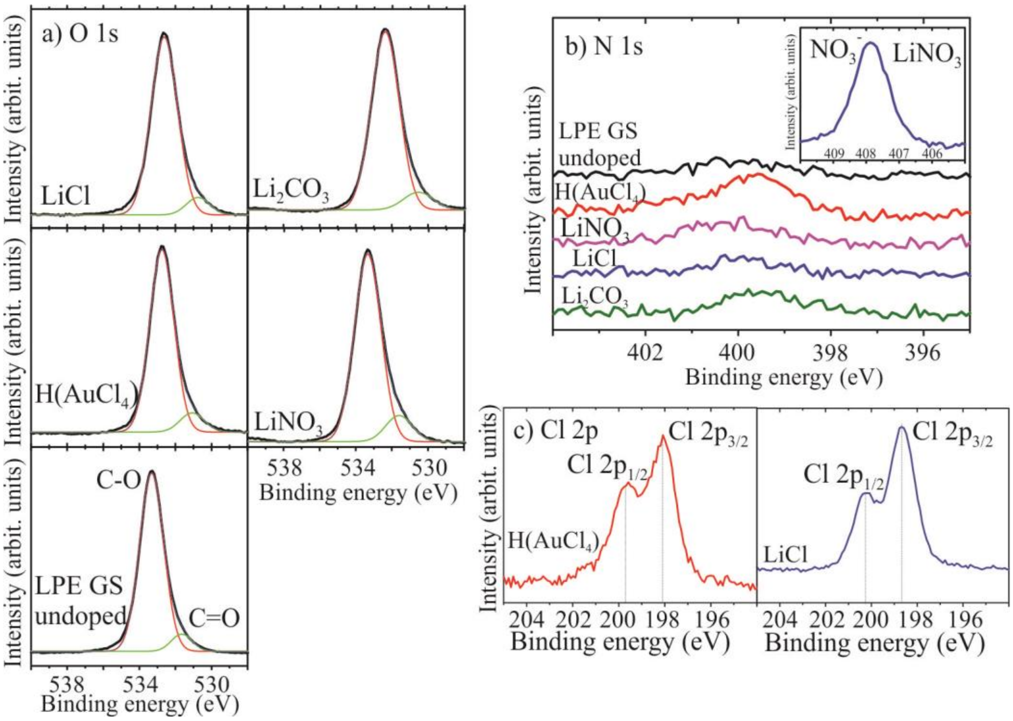

Figure S2: (a) The O 1s core-level XPS spectra for LPE GS undoped and H($AuCl_4$), $LiNO_3$, LiCl and Li_2CO_3 doped films. The O 1s spectra can be deconvoluted in 2 components: C-O (533.6 eV) and C=O (532 eV)[1]. (b) The XPS N 1s spectra (400.0 eV) indicating nitrogen[2,3] presence in undoped and metal-doped films likely due to the residual of NMP/Nitrate peak[4] for $LiNO_3$ doped graphene films at 407.3 eV (inset). c) The XPS Cl 2p spectra for H($AuCl_4$) and LiCl doped LBA GS films. Literature data for Cl $2p_{3/2}$ and Cl $2p_{1/2}$ in the case of H($AuCl_4$) are 198.4 eV[4] and 199.8 eV[5] and for LiCl are 198.8 eV[4] and 200.4[4] eV, respectively.

1. Matković, A. *et al.* Enhanced sheet conductivity of Langmuir-Blodgett assembled graphene thin films by chemical doping. *2D Mater.* **3**, 015002 (2016).
2. Sun, H. *et al.* Binder-free graphene as an advanced anode for lithium batteries. *J. Mater. Chem. A* **4**, 6886–6895 (2016).
3. Thodkar, K. *et al.* Restoring the Electrical Properties of CVD Graphene via Physisorption of Molecular Adsorbates. *ACS Appl. Mater. Interfaces* **9**, 25014–25022 (2017).
4. Naumkin, A. V., Kraut-Vass, A., Gaarenstroom, S. W. & Powell, C. J. NIST X-ray photoelectron spectroscopy database. Available at: https://srdata.nist.gov/xps/EngElmSrchQuery.aspx?EType=PE&CSOpt=Retri_ex_dat&Elm=Li (2019).
5. Syu, J. Y. *et al.* Wide-range work-function tuning of active graphene transparent electrodes via hole doping. *RSC Adv.* **6**, 32746–32756 (2016).

S3: KPFM measurements

CPD of investigated samples was measured by KPFM. It is very convenient technique for investigations of inhomogeneous samples in order to resolve spatial variations of electrical properties at micro- and nano-scale, which is not possible by other techniques. We did KPFM measurements on several (5-6) different 5×5 μm^2 areas on every sample and then averaged them. We observed that the CPD is quite homogeneous and constant despite of inhomogeneity of sample morphology.

Typical example is given in Fig. S3 which depicts topography (left column), CPD maps (middle column) and corresponding CPD histograms (right column) for six different 5×5 μm^2 areas of the LPE graphene film doped with $H(AuCl_4)$. As can be seen, the morphology is quite inhomogeneous. On the other hand, all CPD histograms are characterized with single and well defined peaks positioned at -187 mV, -181 mV, -157 mV, -179 mV, -168 mV, and -162 mV. The average CPD is -172±15 mV. All CPD histograms are shown together in Fig. S4 for clarity. The peaks do not shift appreciably while the deviation is only 15 mV which is less than 10%. Similar procedure was repeated for all investigated samples and the results are summarized in Fig. S5. It depicts measured distributions of CPD for doped LPE GS films for different dopants, with respect to the undoped LPE GS film taken as a reference. Shifting of CPD peaks is clearly resolved demonstrating efficiency of KPFM for studying doping of LPE graphene films.

KPFM was used to calibrate the WF of AFM tips as well. This was done by KPFM measurements on a freshly cleaved HOPG with a well-known work function of 4.6 eV. The average CPD measured on HOPG with Pt covered NSG01/Pt probes was 460 mV. Therefore, the calculated tip work function was $WF_t=WF_{HOPG}+CPD(HOPG)=5.06$ eV. Finally, the sample work function was calculated as $WF_s = WF_t - CPD$, where CPD is measured by KPFM on LPE GS films. In the case of the LPE graphene film doped with $H(AuCl_4)$, the measured WF was 5.232 eV and this is the value presented in Fig. 5(d) of the main manuscript for $H(AuCl_4)$. The same calculations for WF were done for other samples as well.

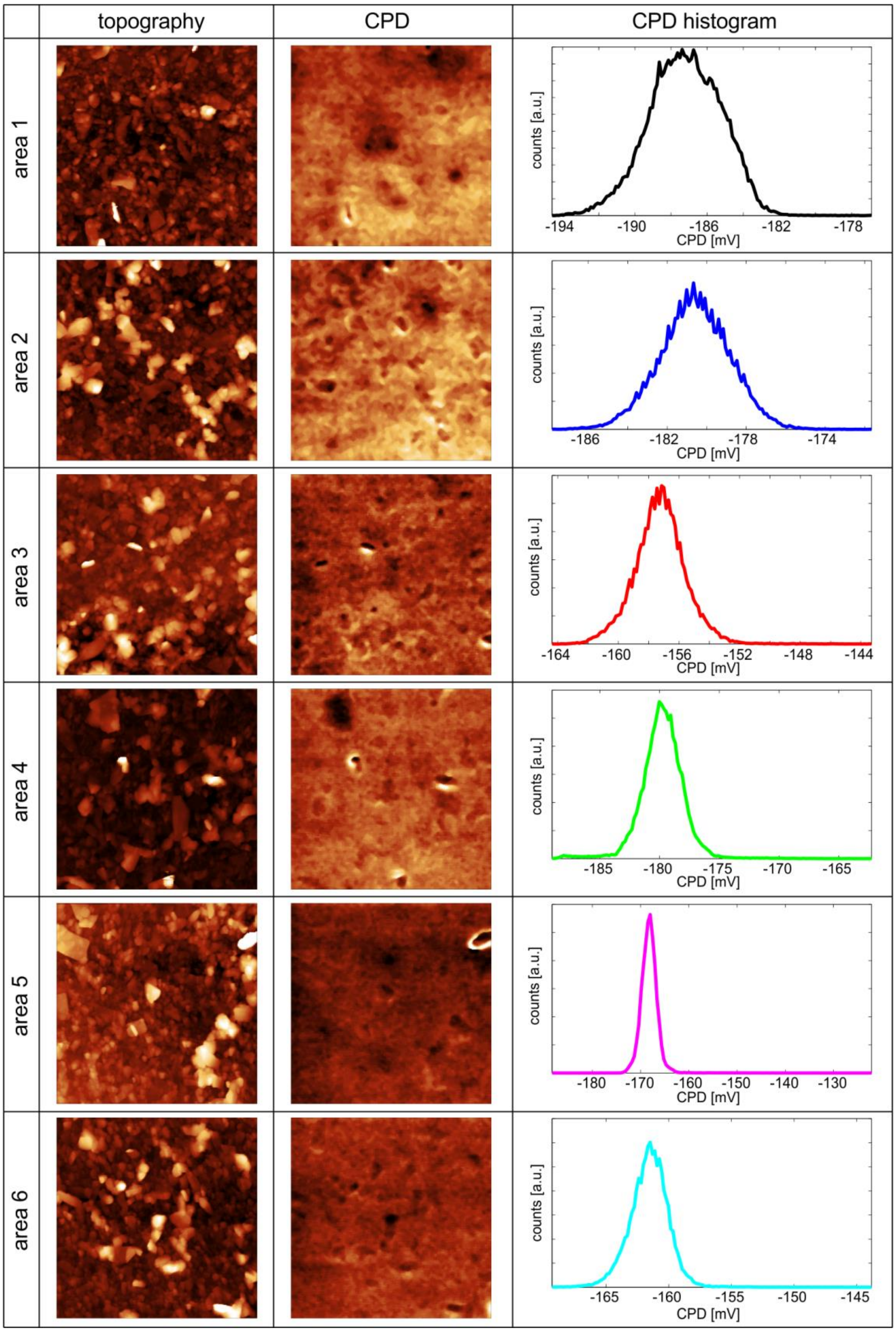

Figure S3: Topography, CPD maps and corresponding CPD histograms for six different 5×5 μm^2 areas of the LPE graphene film doped with $H(AuCl_4)$.

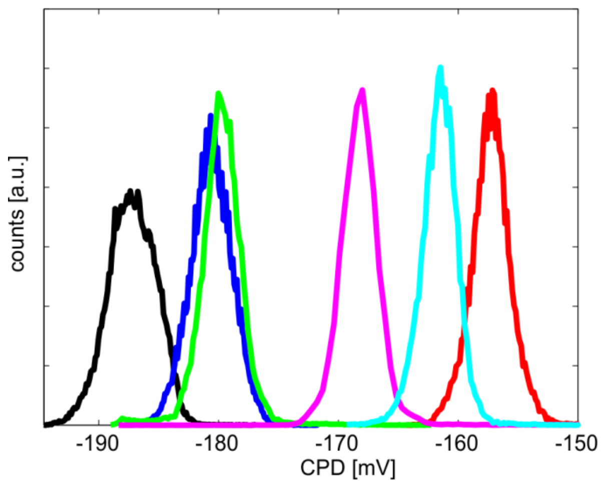

Figure S4: CPD histograms for six different 5×5 μm^2 areas of the LPE graphene film doped with $H(AuCl_4)$ from Figure S3.

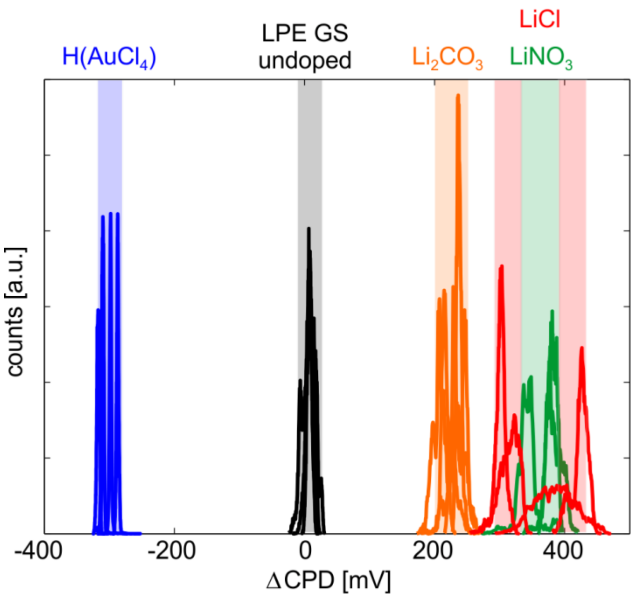

Figure S5: Measured distributions of CPD for doped LPE GS films for different dopants, with respect to the undoped LPE GS film taken as a reference
